# Alpha-6 integrin deletion delays the formation of Brca1/p53-deficient basal-like breast tumors by restricting luminal progenitor cell expansion

**DOI:** 10.1186/s13058-024-01851-4

**Published:** 2024-06-04

**Authors:** Marisa M. Faraldo, Mathilde Romagnoli, Loane Wallon, Pierre Dubus, Marie-Ange Deugnier, Silvia Fre

**Affiliations:** 1 Laboratory of Genetics and Developmental Biology, Institut Curie, INSERM U934, CNRS UMR3215, PSL Research University, 75248 Paris, France; 2 Laboratory of Cell Biology and Cancer, CNRS UMR144, Institut Curie, PSL Research University, 75248 Paris, France; 3 Institut de Recherches Internationales Servier, 91190 Gif Sur Yvette, France; 4 Alacris Theranostics GmbH, 12489 Berlin, Germany; 5 Department of Histology and Pathology, Centre Hospitalier Universitaire de Bordeaux, 33000 Bordeaux, France; 6 BRIC U1312, INSERM, Bordeaux Institute of Oncology, Université de Bordeaux, 33000 Bordeaux, France

**Keywords:** Brca1/p53-deficient tumors, Luminal progenitors, Laminin-binding integrins

## Abstract

**Background:**

The aberrant amplification of mammary luminal progenitors is at the origin of basal-like breast cancers associated with BRCA1 mutations. Integrins mediate cell–matrix adhesion and transmit mechanical and chemical signals that drive epithelial stem cell functions and regulate tumor progression, metastatic reactivation, and resistance to targeted therapies. Consistently, we have recently shown that laminin-binding integrins are essential for the expansion and differentiation of mammary luminal progenitors in physiological conditions. As over-expression of the laminin-binding α6 integrin (Itgα6) is associated with poor prognosis and reduced survival in breast cancer, we here investigate the role of Itgα6 in mammary tumorigenesis.

**Methods:**

We used *Blg-Cre; Brca1*^*F/F*^*; Trp53*^*F/F*^ mice, a model that phenocopies human basal-like breast cancer with BRCA1 mutations. We generated mutant mice proficient or deficient in Itgα6 expression and followed tumor formation. Mammary tumors and pretumoral tissues were characterized by immunohistochemistry, flow cytometry, RT-qPCR, Western blotting and organoid cultures. Clonogenicity of luminal progenitors from preneoplastic glands was studied in 3D Matrigel cultures.

**Results:**

We show that *Itga6* deletion favors activation of p16 cell cycle inhibitor in the preneoplastic tissue. Subsequently, the amplification of luminal progenitors, the cell of origin of Brca1-deficient tumors, is restrained in Itgα6-deficient gland. In addition, the partial EMT program operating in Brca1/p53-deficient epithelium is attenuated in the absence of Itgα6. As a consequence of these events, mammary tumor formation is delayed in Itgα6-deficient mice. After tumor formation, the lack of Itgα6 does not affect tumor growth but rather alters their differentiation, resulting in reduced expression of basal cell markers.

**Conclusions:**

Our data indicate that Itgα6 has a pro-tumorigenic role in *Blg-Cre; Brca1*^*F/F*^*; Trp53*^*F/F*^ mice developing basal-like mammary tumors. In particular, we reveal that Itgα6 is required for the luminal progenitor expansion and the aberrant partial EMT program that precedes the formation of BRCA1 deficient tumors.

**Supplementary Information:**

The online version contains supplementary material available at 10.1186/s13058-024-01851-4.

## Background

The mammary gland undergoes two major morphogenetic events postnatally: the elongation and branching of a ductal tree during puberty and the expansion of the mammary epithelium at pregnancy, accompanied by the generation of secretory alveoli, the milk-secreting units [1]. The regulation of the different steps of postnatal mammary morphogenesis and differentiation is a complex process involving the action of systemic hormones and growth factors [2, 3]. In addition, interaction of epithelial cells with the extracellular matrix (ECM) is essential for mammary gland development and function by modulating cellular responses to soluble factors [4–6].

The mammary epithelium is organized as a bilayer, with a layer of luminal cells lining the ductal or alveolar lumen, and a surrounding layer of basal cells. From mid-pregnancy and during lactation, the luminal cells produce and secrete milk in response to hormone stimulation. The basal (also called myoepithelial) cells express the basal-specific pair of cytokeratins 5/14 (K5 and K14), the transcription factors p63 and Slug, and smooth muscle (SM) contractile proteins like α-SM-actin and SM-myosin. The luminal cells specifically express the cytokeratins 8/18 (K8 and K18). They include a subset of cells expressing the receptors for estrogen (ER) and progesterone (PR) that act as hormone sensors through the production of paracrine signals regulating basal and luminal cell function, and a population devoid of hormone receptor expression (ER/PR-), comprising the luminal progenitors at the origin of the expansion of the mammary epithelium during pregnancy [2]. In recent years, using lineage tracing techniques, numerous studies have revealed that the different mammary lineages originate from embryonic multipotent stem cells. In the postnatal gland, at homeostasis, the three mammary lineages, basal, luminal ER/PR + and luminal ER/PR-, are essentially maintained by their own lineage-restricted unipotent stem/progenitor cells (for review see [1, 7]).

Human breast cancer can be classified into six main molecular subtypes based on transcriptional signatures: luminal A, luminal B, HER2-enriched, normal-like, claudin-low and basal-like [8–10]. The basal-like tumors represent 15–20% of all breast cancers and are characterized by the expression of basal markers such as K5/K14 and p63. They belong to the so-called triple-negative tumors that lack ER and PR and display low levels of HER2 receptor, making them resistant to hormonal or HER2 targeted therapies [11]. Most basal-like tumors display a loss of p53 function [12]. Several studies in the last decade suggest that the distinct breast cancer subtypes originate from different populations of stem and progenitor cells in the mammary hierarchy [7]. Notably, deregulation of luminal ER/PR- progenitors is believed to be at the origin of basal-like breast tumors, particularly those associated with Brca1 mutations [13, 14]. Given the absence of lineage inter-conversion during normal tissue homeostasis, this would imply a switch or a loss of cell identity during tumorigenesis. Accordingly, Brca1 germline mutation has been shown to alter the fate of mammary luminal cells and cause luminal-to-basal transformation [15, 16]. Furthermore, Brca1 loss induces the expression of EMT (epithelial-to-mesenchymal transition) related genes, which are associated with the expansion of cancer stem cells and the formation of basal-like tumors in mice [17].

Among the adhesion systems contributing to the maintenance of the mammary epithelial bilayer, integrins constitute the main cell surface receptors to ECM. They connect the matrix network to the cytoskeleton and trigger biochemical and mechanical signals that, in coordination to soluble factors, control important cell functions [18]. Integrins are heterodimers, composed of an α and a β subunit, and 24 integrin dimers with distinct substrate specificities have been described [19]. Integrins are present in both basal and luminal cells, at all stages of mammary development, and numerous studies have reported their essential roles in controlling mammary cell growth and differentiation [4, 20, 21]. Furthermore, integrin signaling is often deregulated in cancer, affecting the ability of tumor cells to proliferate without control, to become invasive or to survive in adverse conditions [18].

The whole mammary epithelium is surrounded by a specialized ECM, the basement membrane (BM). Laminins are major components of the BM, and the laminin-binding integrin dimers (α3β1, α6β1 and α6β4) are highly expressed in mammary epithelial cells [22]. We have recently reported that laminin-binding integrins have essential roles in the regulation of stem/progenitor cell function during normal mammary gland development [22, 23]. Importantly, α6-integrins (that is, the dimers containing α6-subunit) are involved in the progression of some cancers and in the regulation of normal and cancer stem cells [24]. Here, we explore the role of the α6-integrin in mammary tumorigenesis, using an established mouse model of basal-like cancer, the *Blg-Cre;Brca1*^*F/F*^*;Trp53*^*F/F*^ mice, that develop invasive tumors phenocopying BRCA1-deficient human breast tumors at the molecular and morphological level [14]. Our results reveal that lack of α6-integrin (Itgα6) in luminal progenitors delays the induction of the EMT program that takes place in the early steps of tumorigenesis caused by Brca1 deficiency [15, 17]. Concomitantly, the absence of Itgα6 favors the activation of the p16 cell cycle inhibitor, thereby limiting luminal progenitor expansion in the preneoplastic epithelium. As a result of these alterations, tumor initiation is delayed in Itgα6-deficient Brca1/p53 mutant mice. These results indicate that α6-integrins have a pro-tumorigenic role in basal-like breast cancer and suggest its potential use to dissect the initial steps involved in the establishment of this highly malignant type of breast cancer.

## Methods

### Mice

The generation of *Brca1*^*F/F*^*, Trp53*^*F/F*^* and Itga6 *^*F/F*^ mice has been previously described [25–27]. *BlgCre* transgenic mice were purchased from The Jackson Laboratory. Mice were bred in a mixed 129SV/C57BL6 genetic background. In all experiments, unless specified, *BlgCre*-negative littermates were used as controls. Adult females were assessed by palpation twice a week to monitor tumor appearance. For growth curves, after the first palpation, tumor diameters were measured every 2–3 days and used to calculate tumor volume [28]. Animals were analyzed before the overall tumor burden reached the maximal permitted by the ethics committee (1500 mm^3^). For necrosis analysis, only tumors larger than 1200 mm^3^ were used. Husbandry, supply of animals, as well as maintenance and care in the Animal Facility of Institut Curie (facility license #C75-05-18) before and during experiments fully satisfied the animal’s needs and welfare. All mice were housed and bred in a specific-pathogen-free (SPF) barrier facility with a 12:12 h light–dark cycle and food and water available ad libitum. Mice were sacrificed by cervical dislocation.

### Whole-mount analyses, histology and immunolabeling

For whole-mount Carmine-Alum staining, mammary fat pads were spread onto glass slides, fixed overnight in Methacarn (methanol:chloroform:acetic acid in 6:3:1 proportion) and stained with carmine as previously described [29]. Whole-mount images were acquired with a Leica DFZ420C color video camera in a Leica MZ8 binocular using the LAS (Leica Applications Suite) software. For histological analysis, mammary tissues (tumors or glands) were fixed in 4% paraformaldehyde (PFA) overnight at 4 °C and embedded in paraffin. Sections of 5–7 µm were cut and de-waxed for hematoxylin/eosin staining or immunolabeling. Antigen retrieval was performed by incubating sections in 10 mM citrate buffer pH 5 at 98 °C for 10 min. For cryosections, after fixation in 4% PFA as previously, tumors were incubated in 30% sucrose at 4 °C for 48 h, then frozen on Tissu-Tek (Sakura) and 5–7 µm sections were obtained using a Leica LM1950 cryostat. Sections were incubated with primary antibodies overnight at 4 °C in a humidified chamber then with secondary Alexa-fluor conjugated antibodies and 1 μM DAPI (Sigma) for one hour at room temperature. Finally, sections were mounted in Poly-mount medium (Polysciences), and pictures were obtained in an Upright Spinning Disk Confocal microscope (Roper/Zeiss) with a CoolSnap HQ2 camera. Details of the antibodies used are provided in Additional File 5: Table S1.

### FACS analysis and primary mammary cell preparation

Thoracic and inguinal mammary glands from single virgin females were pooled, dissociated and processed for single-cell suspension and flow cytometry as described elsewhere [30, 31]. Tumors of 1000–1200 mm^3^ were used for dissociation, following the same procedure. For cell sorting, cells were incubated at 4 °C for 20 min with the following antibodies: anti-CD45-APC (clone 30-F11), anti-CD31-APC (clone MEC13.3), anti-mouse Ter119-APC, anti-CD24-PE (clone M1/69), anti-CD49f-APC/Cy7 (clone GoH3), anti-ICAM1-PE/Cy7 (clone YN1/1.7.4), anti-CD29-PE/Cy7 (clone HMb1-1) and anti-CD104-PE/Cy7 (clone 346-11A) all from Biolegend. Labelled cells were analyzed and sorted out using a MoFlo Astrios (Beckman Coulter) or a FACSAria Fusion (BD Biosciences) cell sorters. Sorted luminal progenitor cells (CD31/CD45/Ter119^−^) CD24^high^ CD49f^low^ ICAM^+^ population) were used for single-cell organoid assays and gene and protein expression analysis. Data were analyzed using FlowJo software (v10.10.0).

### Organoid culture and immunostaining

Mammary organoids were derived from fourth-fifth mammary fat pads or tumors as previously described [32]. Briefly, mammary fat pads were minced with scalpels to approximately 1 mm^3^ pieces and digested by incubation for 1 h at 37 °C with 3 mg/ml of collagenase A (Roche) and 1.5 mg/ml of pancreatic trypsin (Sigma) in serum-free Leibowitz L15 medium (Gibco). To eliminate residual single cells such as fibroblast or other stromal cells, four consecutive differential centrifugations at low speed (450 g for 10 s) were performed. The final organoid pellet was mixed with growth-factor reduced Matrigel (BD Biosciences) and seeded in 8-well coverslip bottom chambers (Ibidi) for immunostaining or in 24-well plates for passaging. A suspension containing 50–100 organoids in 30 µl of Matrigel was added to each well. Organoids were cultured for 10–15 days in DMEM/F12 medium containing penicillin/streptomycin, Glutamax, 10 mM HEPES, N2, B27, 100 ng/ml Nrg1 (R&D) 100 ng/ml Noggin (Peprotech/Thermo Fisher Scientific) and 100 ng/ml R-spondin 1 (R&D). The medium was replaced twice a week. To replate organoids, Matrigel was mechanically disrupted by pipetting up and down in ice-cold PBS with a P1000 pipette. After centrifugation at 450 g for 5 min, organoid pellets were resuspended in fresh Matrigel and plated as described above. For single cell organoids, 15,000 sorted cells were resuspended in a 20 µl drop of Matrigel and cultured for 12–14 days in 24-well plates as described above.

For immunostaining, organoids were fixed in 4% PFA at RT for 1 h and permeabilized with 1% Triton-X-100. Non-specific epitopes were blocked by incubating organoids in 2% BSA, 5% FBS and 0.25% Triton-X-100 for 1 h. Antibodies were diluted in blocking solution and incubated overnight (primary antibodies) and 5 h (secondary antibodies) at RT. For EdU incorporation assays, 10 µM EdU was added to the organoids 2 h before fixation. EdU was revealed after organoid immunostaining using a Click-iT EdU Imaging kit (Invitrogen) following manufacturer’s instructions. After staining, organoids were kept in 50% glycerol in PBS at 4 °C until imaging using an Inverted Spinning Disk Wide Confocal Microscope CSU-W1 (Roper/Nikon/Gattaca) and a sCMOS BSI camera (Nikon).

### RNA extraction and RT-qPCR

Total RNA was isolated from sorted cells using RNeasy Microkit (Qiagen). To avoid eventual DNA contamination, purified RNA was treated with RNAse-free DNAse (Qiagen). RNAs were reverse-transcribed using SuperScript IV Vilo (Thermo Fisher Scientific). Quantitative PCR was performed using the QuantiNova SYBR Green PCR Kit (Qiagen) on a LightCycler 480 real-time PCR system (Roche). The values obtained were normalized to *Gapdh* levels. The primers used for RT-qPCR analysis were purchased from SABiosciences/Qiagen or designed using Oligo 6.8 software (Molecular biology Insights) and synthesized by Eurogentec. Primers used in this study are listed in Additional File 6: Table S2.

### Western blot analysis

Sorted cell pellets were resuspended in hot 1.5 × Laemli buffer, vortexed and boiled for 5 min. Samples were run on NuPAGE Novex 4–12% Bis Tris gels (Life Technologies/Invitrogen) and transferred onto nitrocellulose. Membranes were incubated with 5% BSA in TBS containing 0.1% Tween 20 (TBST) for 1 h at room temperature and with primary antibodies overnight at 4 °C. The primary antibodies used are indicated in Additional File 5: Table S1. Secondary antibodies coupled to horseradish peroxidase were from Cell Signalling Technology. Detection was performed by chemiluminiscence (Super signal West Pico+, Pierce) using a ChemiDoc MP imaging system (Bio-Rad).

### In silico gene expression analysis

Illumina RNAseq gene expression data of 1247 breast cancer cases in TCGA cohorts classified according to PAM50 signature were downloaded from Xena Browser (https://xenabrowser.net). mRNA expression levels of *ITGA6* and *ITGA3* were imported as normalized log^2^ values and visualized as box and whisker plots using Prism (GraphPad) v10.2.2.

### Statistical analysis of the data

At least n = 3 animals were used for each experiment. Statistical tests and further graphs were prepared in Prism (GraphPad) v10.2.2. For survival curves, a long-rank (Mantel-Cox) test was used. All graphs show mean ± standard error of the mean (SEM). Except when specifically indicated, differences between groups were assessed with Student’s t test with two-tailed distribution and unequal variance (Welch’s correction). The significance threshold was *p* < 0.05. * indicates *p* < 0.05, ** indicates *p* < 0.01, *** indicates *p* < 0.001, and **** indicates *p* < 0.0001.

## Results

### Tumor formation is delayed in basal-like Brca1/p53-deficient tumors lacking *Itga6*

**Fig. 1:**
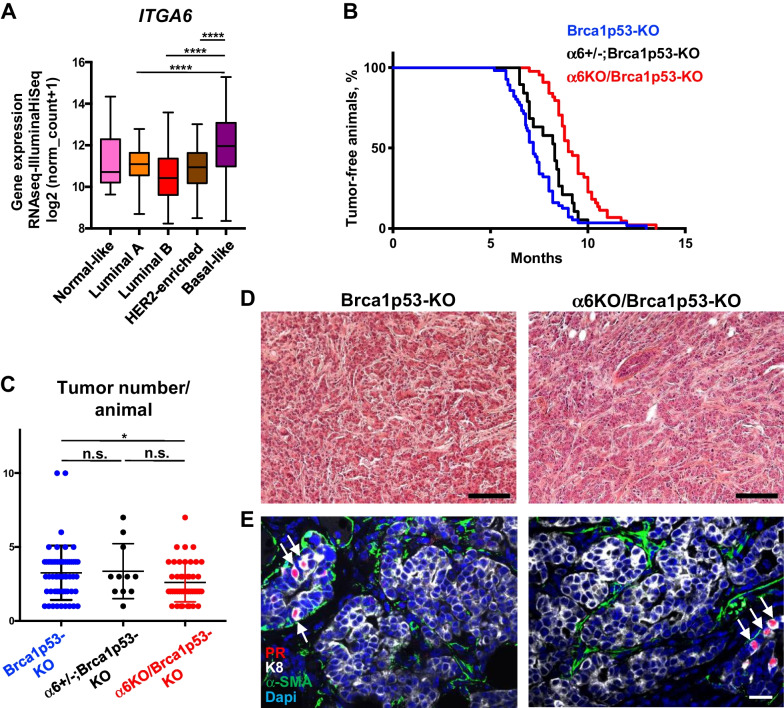
Tumor formation is delayed in basal-like Brca1/p53-deficient tumors lacking *Itga6*.** A** In silico analysis of *ITGA6* mRNA expression in human breast cancer subtypes classified by the PAM50 signature: Normal-like (n = 8), Luminal A (n = 231), Luminal B (n = 127), HER2-enriched (n = 58), and basal-like (n = 98). **B** Kaplan–Meier tumor-free survival curve of Brca1p53-KO (n = 56) α6 ± ;Brca1p53-KO (n = 19) and α6KO/Brca1p53-KO (n = 44) females. *P* = 0.13 (Brca1p53-KO vs. α6 ± ;Brca1p53-KO); *P* = 0.0001 (Brca1p53-KO vs. α6KO/Brca1p53-KO); *P* = 0.001 (α6 ± ;Brca1p53-KO vs. α6KO/Brca1p53-KO). **C** Number of mammary tumors per mouse in Brca1p53-KO (n = 55) α6 ± ;Brca1p53-KO (n = 11) and α6KO/Brca1p53-KO (n = 43) females. **D, E** Histological analysis of Brca1p53-KO and α6KO/Brca1p53-KO tumors. **D** Hematoxylin/eosin staining. **E** Immunofluorescent staining with anti-PR (red), anti-K8 (white) and anti α-SMA (green) antibodies. Nuclear DAPI staining is shown in blue. In the tumors, α-SMA staining is mostly restricted to tumor fibroblast. White arrows indicate the presence of normal ductal luminal cells positively stained for PR. Scale bar: 100 µm (**D**) and 20 µm (**E**). In **A**, **C**: *P < 0.05, ****P < 0.0001, n.s., non-significant

We have recently shown that laminin-binding integrins (containing the α3- and α6- integrins subunits, encoded by the *Itga3* and *Itga6* genes, respectively) are essential for the regulation of mammary stem/progenitor cell function and for mammary development during pregnancy and lactation [22, 23]. To study their role in tumorigenesis, we first interrogated the TCGA database comprising the main molecular subtypes of breast cancer, for the expression of *ITGA3* and *ITGA6* transcripts. Interestingly, *ITGA6* levels were significantly higher in basal-like tumors when compared with the other breast cancer subtypes, while *ITGA3* levels were lower in this group (Fig. 1A, Additional File 1: Fig. S1A). This prompted us to investigate whether Itgα6 plays a role in the formation of basal-like breast cancer. To this purpose, we used *Blg-Cre; Brca1*^*F/F*^*;Trp53*^*F/F*^ mice (hereafter referred to as Brca1p53-KO), an established mouse model of basal-like cancer closely resembling BRCA1-deficient human tumors [14]. In these mice, the Blg (beta-lactoglobulin) promoter targets Cre recombinase expression to the ER/PR- luminal progenitor population, inducing the specific *Brca1* and *Trp53* gene deletion in these cells [14, 23]. Brca1p53-KO mice were crossed with *Itga6*^*F/F*^ mice, to obtain *Blg-cre;Brca1*^*F/F*^*; Trp53*^*F/F*^*;Itga6*^*F/WT*^ and *Blg-cre;Brca1*^*F/F*^*;Trp53*^*F/F*^*;Itga6*^*F/F*^ mice (hereafter referred to as α6 ± ;Brca1p53-KO and α6KO/Brca1p53-KO, respectively) and cohorts of mice of the three genotypes were monitored for tumor formation (Fig. 1B). All mice developed mammary tumors within a period of 13.5 months. Brca1p53-KO females developed palpable mammary tumors with a mean latency of 7.2 months. A slight, non-statistically significant delay in tumor onset was observed in α6 ± ; Brca1p53-KO females (mean latency of 8.3 months). Strikingly, homozygous *Itga6* deficiency induced a significant delay in tumor formation, with a mean latency of 9 months in α6KO/Brca1p53-KO mice (Fig. 1B). Furthermore, the number of tumors per animal was not affected in a6 ± ; Brca1p53-KO but was significantly reduced in α6KO/Brca1p53-KO females, when compared to Brca1p53-KO females (Fig. 1C). The α6KO/Brca1p53-KO (α6-deficient) females were then used for further analysis and compared to female mice expressing normal levels of Itgα6 throughout the study. Small (< 5 mm diameter) α6KO/Brca1p53-KO mammary tumors had histological characteristics comparable to Brca1p53-KO small tumors and were mainly classified as invasive ductal carcinoma of non-special type (IDC-NST) (Fig. 1D). In larger tumors (≥ 1200 mm^3^), extended or multifocal necrotic areas were evidenced in half of Brca1p53-KO tumors but not in α6KO/Brca1p53-KO tumors (Additional File 1: Fig. S1B). In both cohorts, the tumors were mostly positive for the luminal marker Keratin 8 (K8) but devoid of progesterone receptor (PR) as previously described for Brca1p53-KO tumors (Fig. 1E; [14, 25]). PR was, however, detected in normal-looking ducts adjacent to the tumors, as well as in tumor-free mammary tissues (Fig. 1E, Additional File 1: Fig. S1C). The absence of the Itgα6 subunit in the majority of tumor cells of α6KO/Brca1p53-KO mice was confirmed by immunostaining (Additional File 1: Fig. S1D). As we previously reported in normal mammary glands of *Blg-cre; Itga6*^*F/F*^ mice, most α6-deficient tumor cells also lack β4 integrin at the membrane (Additional File 1: Fig. S1D; [23]).

These results indicate that the lack of Itgα6 delays the formation of basal-like mammary tumors in Brca1/p53 deficient mice without inducing major changes in their global histopathological phenotype.

### Itga6 deletion alters the differentiation of Brca1/p53-deficient tumor cells

**Fig. 2:**
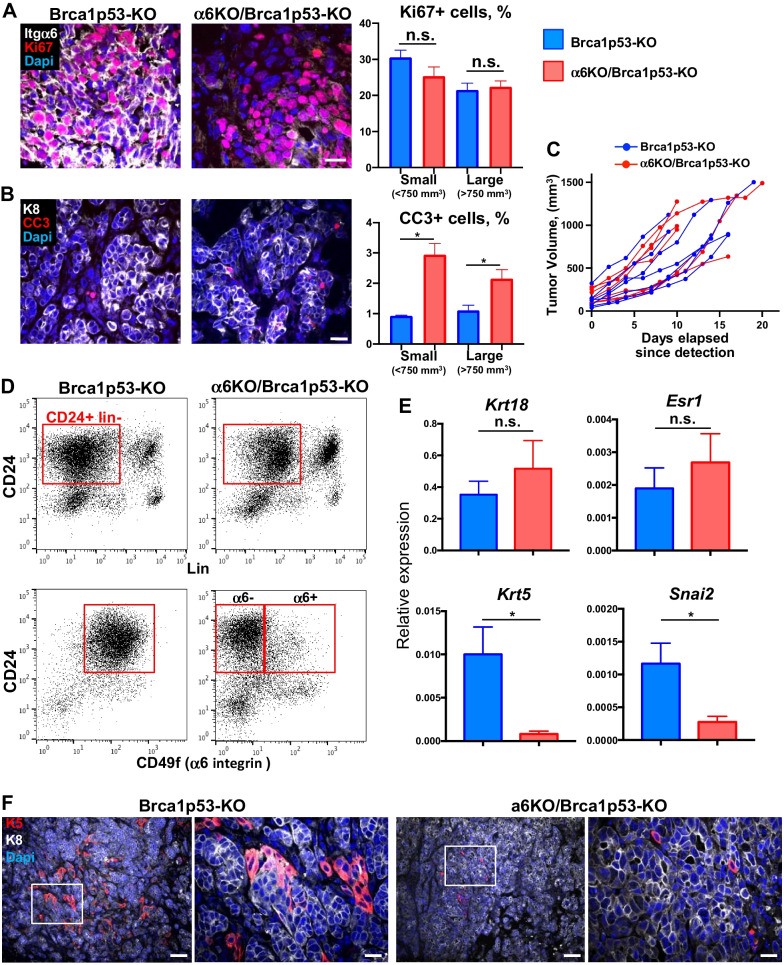
Effect of *Itga6* deletion on growth and differentiation of Brca1/p53-deficient tumors. **A** Immunofluorescent staining with anti-Itgα6 (white), and anti-Ki67 (red) antibodies. The graph shows the percentage of Ki67 + cells (mean ± SEM) in small (< 750 mm^3^; n = 8 animals per genotype) and large (> 750 mm^3^; n = 7 animals per genotype) tumors. **B** Immunofluorescent staining with anti-K8 (white), and anti-cleaved caspase 3 (CC3, red) antibodies. The graph shows the percentage of CC3 + cells (mean ± SEM) in small (< 750 mm^3^; n = 4 animals per genotype) and large (≥ 750 mm^3^; n = 4 animals per genotype) tumors. **A** and **B**: Nuclear DAPI staining is shown in blue. Scale bar: 20 µm. **C** Tumor growth curves of Brca1p53-KO (n = 7) and α6KO/Brca1p53-KO (n = 7) tumors. The difference between groups is not significant (Kruskal–Wallis test). **D** Upper panels: Representative FACS analysis of a Brca1p53-KO and a α6KO/Brca1p53-KO tumor with CD24 and Lin (CD31/CD45/Ter-119) antibodies. The red rectangle shows the CD24^+^ Lin^−^ population sorted for gene expression analysis. Lower panels: Representative FACS analysis of tumors with CD24 and CD49f (α6-integrin) antibodies showing depletion of surface Itgα6 in most epithelial cells of the α6KO/Brca1p53-KO tumor.** E** RT-qPCR analysis of the CD24^+^ Lin^−^ tumor cell population from Brca1p53-KO (n = 5) and α6KO/Brca1p53-KO (n = 5) animals (mean ± SEM). **F** Representative immunofluorescent staining with anti-K5 (red) and anti-K8 (white) antibodies. Magnification of the white rectangle is shown in the right panels of Brca1p53-KO and α6KO/Brca1p53-KO tumors. Nuclear DAPI staining is shown in blue. Scale bar: 75 µm (left panels for each tumor) and 20 µm (magnification in the right panels). In **A**, **B**, **E** *P < 0.05, n.s., non-significant

To investigate if tumor growth was affected by the lack of Itgα6, we first analyzed cell proliferation by immunostaining with an anti-Ki67 antibody. Similar proliferation rates were found in α6-deficient and α6-proficient tumors, independently of the tumor size (Fig. 2A). Likewise, tumor organoid cultures presented analogous proliferation rates in both groups, even after several organoid passages, as shown in EdU-incorporation assays (Additional File 2: Fig. S2A, B). Integrin activation has been involved in cell survival in different tissues [33]. We then assessed apoptosis by cleaved-caspase-3 (CC3) staining and found that apoptosis rates were low and only slightly increased in α6KO/Brca1p53-KO tumors (around 2–3% of CC3 + tumor cells; Fig. 2B). In agreement with these data, systematic measurement of tumor size after initial palpation showed that tumor growth was not significantly affected by the absence of *Itga6* (Fig. 2C).

We subsequently isolated tumor cells (CD24^+^Lin^−^) by flow cytometry and performed gene expression analyses for deeper characterization (Fig. 2D, upper panels; Additional File 2: Fig. S2C). As previously found by immunostaining of tumor sections (Additional File 1: Fig. S1D), flow cytometry and RT-qPCR analysis further confirmed the reduction of *Itga6* expression in α6KO/Brca1p53-KO tumor cells (Fig. 2D, lower panels; Additional File 2: Fig. S2D). *Trp53* and *Brca1* genes, although similarly expressed in the tumor cells of both groups, were significantly down-regulated when compared to wild-type luminal cells (Additional File 2: Fig. S2D). Expression of the luminal genes *Krt18*, *Esr1* and *Cdh1* (encoding respectively for cytokeratin 18, estrogen receptor α and E-cadherin) was not significantly changed in α6KO/Brca1p53-KO tumors (Fig. 2E, Additional File 2: Fig. S2E). Similarly, the level of *Tnfsf11 and Tnfrsf11a* encoding respectively for the secreted factor RANKL and its receptor, RANK, involved in the oncogenesis of Brca1 mutation-driven breast cancer, was not altered in the absence of Itgα6 (Additional File 2: Fig. S2E; [34]). In contrast, the basal genes *Krt5* (encoding for cytokeratin 5) and *Snai2* (encoding for the transcription factor Slug) were significantly down-regulated in α6-deficient tumors (Fig. 2E). Diminished expression of Krt5 was confirmed by immunostaining (Fig. 2F).

Collectively, these results indicate that lack of Itgα6 does not affect tumor growth but alters the differentiation of Brca1/p53-deficient mammary tumor cells.

### Itga6 deletion impairs luminal progenitor cell activity in Brca1/p53-deficient preneoplastic glands

**Fig. 3:**
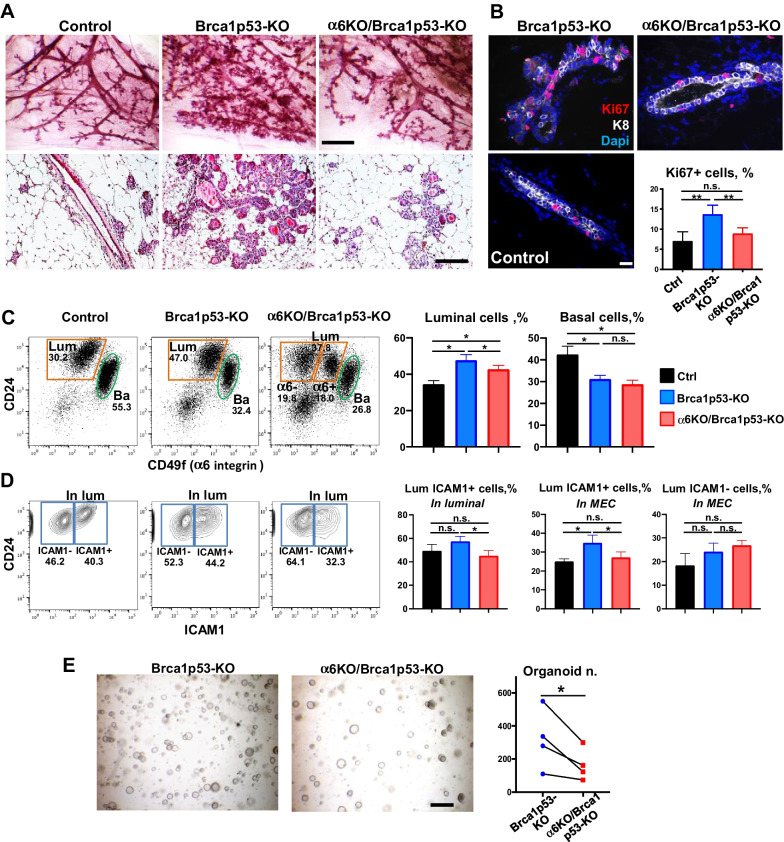
Analysis of the preneoplastic gland of Brca1/p53-deficient mice. **A** Representative microphotographs of mammary glands from 5-month-old virgin control, Brca1p53-KO and α6KO/Brca1p53-KO mouse. Upper panels: fragments of glands stained with Carmine in whole-mount. Lower panels: Hematoxylin and Eosin staining of gland sections. Scale bar: 1 mm (upper panels) and 100 µm (lower panels). **B** Immunofluorescent staining with anti-K8 (white), and anti-Ki67 (red) antibodies. Nuclear DAPI staining is shown in blue. Scale bar: 20 µm. The graph shows the percentage of Ki67 + cells (mean ± SEM) obtained from five animals per group. **C, D** Representative FACS analysis of mammary glands from 5-months-old virgin mouse. **C** separation of basal (green) and luminal (orange) populations in control and Brca1/p53-deficient mammary glands. Note that in the α6KO/Brca1p53-KO gland, a fraction of luminal cells are depleted of Itgα6 expression. Right: graphs showing the percentage of luminal and basal cells in the Lin- population, in 7 independent cell sorting experiments (mean ± SEM). **D** Analysis of ICAM1 expression gated in the luminal cells. Right: graphs showing the percentage of luminal ICAM1^+^ cells in the luminal gate and the ICAM1 + and ICAM1- in the total MEC population in 7 independent cell sorting experiments (mean ± SEM). **E** Representative microphotographs of organoids formed by sorted luminal ICAM1^+^ cells after 12 days of culture. Scale bar: 250 µm. Right: graph showing the number of organoids per well obtained in 4 independent cell sorting experiments. In **B**-**E** *P < 0.05, **P < 0.01, n.s., non-significant

To further characterize the early events leading to the observed delay in tumorigenesis in α6KO/Brca1p53-KO mice, we analyzed the mammary tissue of 4–5 month-old females, before the appearance of tumors (here after referred to as preneoplastic glands). In contrast to wild-type glands, comprising essentially mammary ducts with limited branching, the glands of Brca1p53-KO females were hyper-branched and contained a high number of alveolar-like structures, as assessed by Carmine staining and histological analyses (Fig. 3A). This phenotype was previously reported in similar Brca1-deficient mouse models [35, 36]. However, in α6KO/Brca1p53-KO mice this aberrant mammary phenotype was less pronounced (Fig. 3A). Consistently, proliferation rates were increased in Brca1p53-KO mammary epithelium but were close to normal in α6-deficient glands (Fig. 3B).

Flow cytometry analysis was performed with preneoplastic glands, to separate basal and luminal mammary cells as previously reported [30, 31]. Compared to control glands, luminal cells were amplified in Brca1/p53 mutants, and this increase was clearly more pronounced in α6-expressing mice, at the expense of the basal population which was decreased in both mutant mice relative to control glands (Fig. 3C). In addition, we could confirm that Itgα6 was depleted in a substantial fraction of luminal cells in the preneoplastic tissue of α6KO/Brca1p53-KO mice (Fig. 3C). Itgα6 chain can bind to β1 or β4 chains to form α6β1 and α6β4 laminin-receptor dimers. In the luminal cells of α6KO/Brca1p53-KO glands, Itgβ4 surface levels were decreased when compared to Brca1p53-KO mice, while basal cells displayed similar Itgβ4 levels in both groups (Additional File 3: Fig. S3A). No differences were detected in Itgβ1 expression between both groups in luminal and in basal cells (Additional File 3: Fig. S3A).

We have previously reported that in wild-type glands, ICAM1 expression discriminates two luminal cell populations, consisting of highly clonogenic progenitors (ICAM1 + , mostly ER/PR-) and poorly clonogenic, mature (ICAM1-, ER/PR +) luminal cells [37, 38]. Similarly, two populations were separated by ICAM1 in Brca1/p53 mutant tissues: ICAM1 + (hereafter referred to as luminal progenitors or LP) and ICAM1- (hereafter referred to as luminal mature or LM) which is enriched in cells expressing the hormone receptor ERα (Fig. 3D, Additional File 3: Fig. S3B). The Blg promoter has been reported to specifically target luminal progenitor cells [14, 23] and we confirmed these results in our mice, showing that *Cre* expression was found predominantly in ICAM1 + LP (Additional File 3: Fig. S3C). Accordingly, FACS analysis revealed that, in α6KO/Brca1p53-KO glands, integrin depletion is mostly detected in the ICAM1 + luminal progenitor population and this was confirmed by RT-qPCR analysis (Additional File 3: Fig. S3D, E). Furthermore, immuno-staining of α6KO/Brca1p53-KO mammary gland sections showed that the PR + cells retain expression of Itgα6 at their surface, indicating that mature luminal cells are not targeted for integrin deletion by BlgCre (Additional File 3: Fig. S3F).

Within the luminal cell population, the proportion of ICAM1 + LP was not significantly changed in Brca1/p53 mutants when compared to control animals, confirming previously reported results in a similar mouse model (Fig. 3D; [14]). However, when quantified over the proportion of total mammary epithelial cells (MECs), the percentage of ICAM1 + LP was increased in Brca1p53-KO glands and, of interest, it was rescued to control numbers in α6-deficient glands (Fig. 3D). Thus, in α6KO/Brca1p53-KO mice, the observed increase in luminal cells (Fig. 3C), appears to derive from an over-representation of ML cells, that indeed show a trend to increase, although without reaching statistical significance. Gene expression analysis of sorted mutant luminal progenitors confirmed a decrease in *Brca1* and *Trp53* transcripts compared to control cells, while the expression of known markers of these cells (i.e. *Krt18*, *Elf5, Csn2*) was similar to control (Additional File 3: Fig. S3E). Furthermore, we cultured sorted luminal progenitors in Matrigel as 3D organoids [32] and found that their ability to form organoids, was severely impaired upon Itgα6 deletion (Fig. 3E).

Altogether these results indicate that deletion of *Itga6* inhibits the expansion of luminal progenitors induced by Brca1/p53 loss and impairs their clonogenicity during the preneoplastic steps taking place before tumor development.

### Itga6 deletion inhibits the ectopic expression of basal/EMT-like genes in Brca1/p53-deficient luminal progenitors

**Fig. 4:**
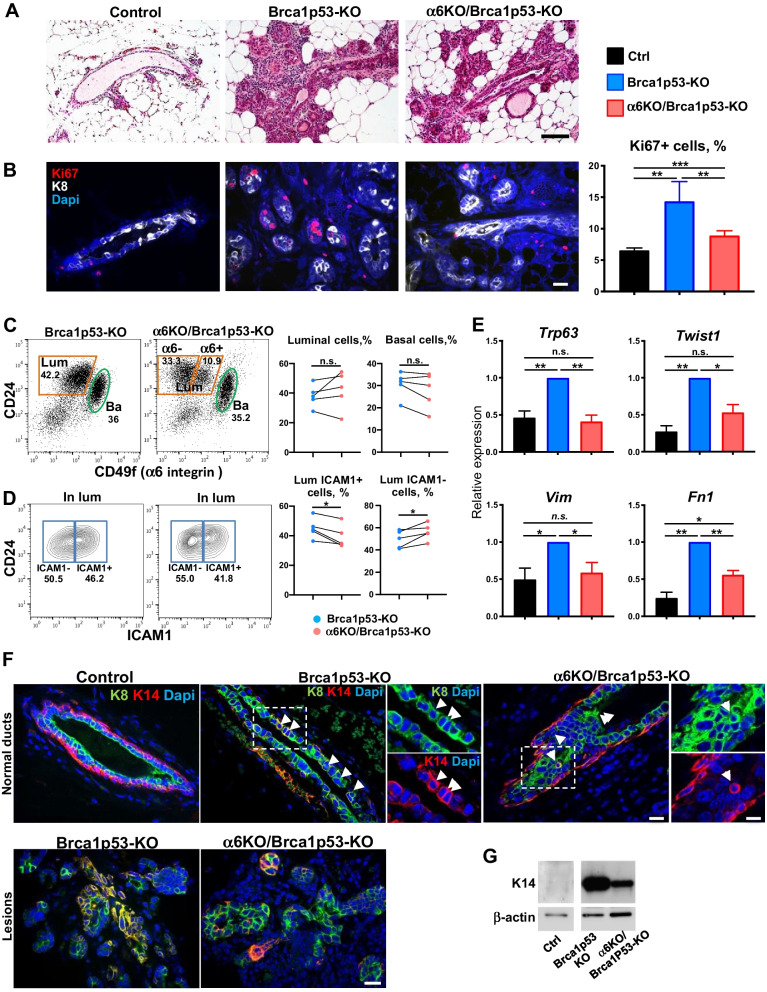
The aberrant expression of basal/EMT-like genes in luminal progenitors of Brca1/p53-deficient mice is reduced by *Itga6* deletion. **A** Hematoxylin and Eosin staining of juxta-tumoral mammary gland sections from Brca1p53-KO and α6KO/Brca1p53-KO mice (bearing a tumor in another gland) and a control littermate. Scale bar: 100 µm. **B** Immunofluorescent staining with anti-K8 (white), and anti-Ki67 (red) antibodies. Nuclear DAPI staining is shown in blue. Scale bar: 20 µm. The graph shows the percentage of Ki67 + cells (mean ± SEM) obtained from 4 controls, 6 Brca1p53-KO and 6 α6KO/Brca1p53-KO mice. **C**, **D** Representative FACS analysis of juxta-tumoral glands from Brca1p53-KO and α6KO/Brca1p53-KO mice. **C** separation of basal (green) and luminal (orange) populations. Right: graph showing the percentage of basal and luminal cells in the Lin- population in 5 independent cell sorting experiments. **D** Analysis of ICAM1 expression gated in the luminal cells. Right: graph showing the percentage of luminal ICAM1^+^ and ICAM1- cells relative to the total luminal population in five independent cell sorting experiments. **E** RT-qPCR analysis of the ICAM1 + LP from Brca1p53-KO and α6KO/Brca1p53-KO juxta-tumoral glands and control glands (5 animals per group) The graphs present mean ± SEM. **F** Immunofluorescent staining with anti-K8 (green), and anti-K14 (red) antibodies of control normal gland and normal-looking ducts (upper panels), or of mammary hyperplasic lesions (lower panels) developed in Brca1p53-KO and α6KO/Brca1p53-KO mice. In normal ducts, dashed rectangles indicate magnifications shown in the right panels. Note the presence of numerous cells co-expressing K8 and K14, indicated by white arrowheads in normal ducts, or marked in yellow in lesions (lower panels). Nuclear DAPI staining is shown in blue. Scale bar: 20 µm (12 µm in magnifications). **G** Western blot analysis of ICAM1 + LP isolated from Brca1p53-KO and α6KO/Brca1p53-KO juxta-tumoral glands. Control cells are shown for comparison. In **B**–**E**, *P < 0.05, **P < 0.01, ***P < 0.001, n.s., non-significant

We then explored tumor progression, by analyzing the non-tumoral glands from mice harboring palpable tumors (hereafter referred to as juxta-tumoral glands). At this stage, mammary gland morphology was often perturbed, with hyperplastic areas developing in both Itgα6-deficient and -proficient glands (Fig. 4A). Consistently, proliferation rates were increased in the glands of both Brca1 mutant groups when compared with the normal epithelium; however, the increase in proliferation was attenuated in α6KO/Brca1p53-KO glands (Fig. 4B). Enhanced proliferation was also detected in organoids derived from tissue fragments of mutant mice, indicating an epithelium cell-autonomous phenotype (Additional File 4: Fig. S4A). Decreased expression of Itgα6 was confirmed in the luminal cells of α6KO/Brca1p53-KO glands by flow cytometry and RT-qPCR (Fig. 4C; Additional File 4: Fig. S4B). Of note, the fraction of luminal cells depleted of Itgα6 was significantly higher in juxta-tumoral glands (8–10-month-old females) than in preneoplastic tissue (4–5-months-old females) in α6KO/Brca1p53-KO mice, probably due to the increased expression of the Blg promoter with age, as previously reported (Additional File 4: Fig. S4C; [14]). The proportion of luminal and basal cells was not significantly different in Brca1p53-KO and α6KO/Brca1p53-KO juxta-tumoral glands (Fig. 4C). However, within the luminal cell population, the proportion of ICAM1 + LP was still decreased in Itgα6-deficient glands, similarly to what observed in the preneoplastic gland (Fig. 4D).

Before tumor formation, Brca1/p53-deficient luminal cells have been reported to acquire some basal-like traits and to express EMT-related genes [17, 39]. Consistently, in Brca1p53-KO luminal progenitors, we found a significant increase in several basal transcripts (such as *Trp63*, encoding for the transcription factor p63) and EMT-related genes, such a *Fn1* (encoding for the ECM protein fibronectin), *Vim* (encoding for the cytoskeletal protein vimentin) as well as the transcription factor *Twist1*, compared to control cells (Fig. 4E). Notably, in the α6KO/Brca1p53-KO luminal progenitors, expression of these genes was reduced when compared to the Brca1p53-KO cells (Fig. 4E). In addition, the basal marker cytokeratin 14 (K14) was readily detected in luminal (K8 +) cells of Brca1p53-KO epithelium, both in normal-looking ducts detected at this stage and in the pretumoral lesions (Fig. 4F). Interestingly, such cells co-expressing luminal and basal markers (K8 + /K14 +) were rarely found in α6KO/Brca1p53-KO epithelium, just like in nascent mammary lesions of juxta-tumoral glands (Fig. 4F). Western blot analysis performed with extracts of sorted luminal progenitor cells confirmed K14 differential expression in Brca1p53 mutant cells and the partial rescue of its expression upon α6 knock-out (Fig. 4G).

Taken together, these results indicate that Itgα6 contributes to the acquisition of the expression of basal/EMT-associated genes in Brca1/p53-deficient luminal progenitors prior to tumor formation. It is tempting to speculate that cells with mixed luminal/basal and EMT features might represent tumor cells of origin or the cells responsible for tumor growth and progression. If that was the case, the decrease of such cells in α6 KO mice might reflect the delay in tumor formation observed in these compound mice.

### Lack of *Itga6* favors the induction of p16 caused by Brca1 deficiency in preneoplastic glands

**Fig. 5:**
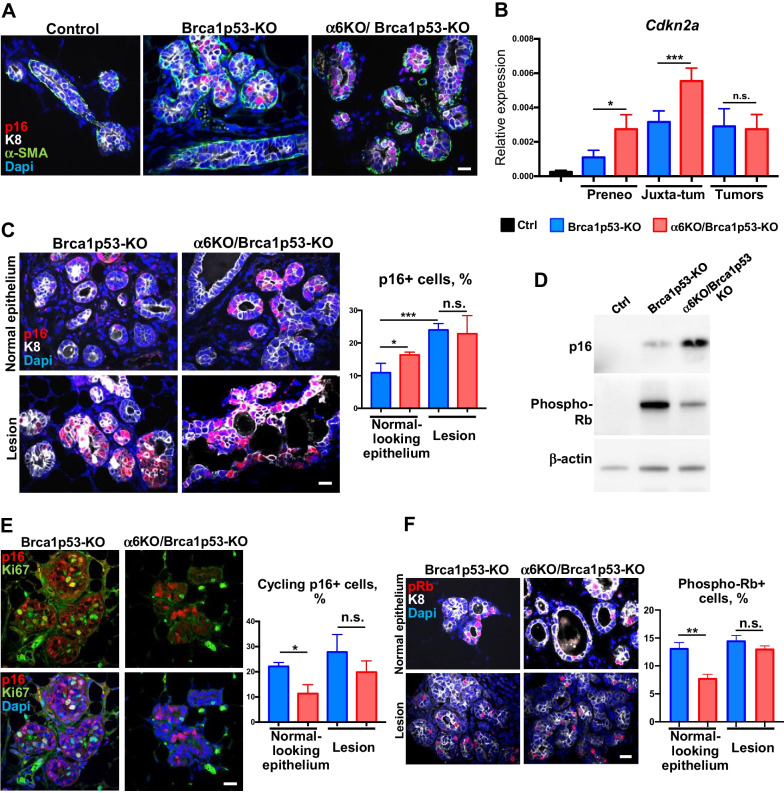
Induction of the cell cycle inhibitor p16 in the Brca1/p53-deficient preneoplastic glands. **A** Immunofluorescent staining with anti-p16 (red), anti-K8 (white) and anti α-SMA (green) antibodies. Nuclear DAPI staining is shown in blue. Scale bar: 20 µm. **B** RT-qPCR analysis of *Cdkn2a* expression (coding for the p16 protein) in ICAM1 + LP cells (n = 4) and CD24 + tumor cells (n = 3) from Brca1p53-KO and α6KO/Brca1p53-KO mice at different stages. Control ICAM1 + LP values are shown for comparison (n = 4). **C** Immunofluorescent staining with anti-p16 (red) and anti-K8 (white) antibodies. Nuclear DAPI staining is shown in blue. Scale bar: 20 µm. The graph shows the percentage of p16 + cells (mean ± SEM) obtained from 3–4 animals per group. **D** Western blot analysis of luminal progenitor cells isolated from Brca1p53-KO and α6KO/Brca1p53-KO juxta-tumoral glands. Cells isolated from control littermate females are shown for comparison. **E** Immunofluorescent staining with anti-p16 (red), and anti-Ki67 (green) antibodies. Nuclear DAPI staining is shown in blue. Scale bar: 20 µm. The graph shows the fraction of p16 + that are also Ki67 + (mean ± SEM) obtained from 3–4 animals per group. **F** Immunofluorescent staining with anti-phospho-Rb (red), and anti-K8 (white) antibodies. Nuclear DAPI staining is shown in blue. Scale bar: 20 µm. The graph shows the percentage of phospho-Rb + luminal (K8 +) cells (mean ± SEM) obtained from 4 animals per group. In **B**, **C**, **E**, **F**, *P < 0.05, **P < 0.01, ***P < 0.001, n.s., non-significant

Among invasive breast cancer, basal-like tumors are characterized by the high expression of the cell cycle inhibitor p16 at the transcriptional level [40]. Moreover, the presence of a p16-expressing luminal cell population in preneoplastic *Blg-Cre;Brca1*^*F/F*^*;Trp53*^*F/F*^ mammary tissue has been recently reported [41]. In agreement with these recent findings, we detected p16 expression in luminal cells (K8 + /α-SMA) from 5-month-old Brca1 mutant mice, but not in control mice (Fig. 5A). In Brca1p53-KO mice, expression of the *Cdkn2a* gene (encoding for the p16 protein) in luminal progenitors progressively increased from preneoplastic (predominantly normal-looking epithelium) to juxta-tumoral glands (presenting some hyperplastic lesions), but not in fully grown tumors (Fig. 5B). Interestingly, in α6KO/Brca1p53-KO mutants, luminal progenitors displayed higher levels of *Cdkn2a* transcripts than Itgα6-proficient cells, while no difference was detected between both groups at the tumor stage (Fig. 5B). We then investigated the spatial distribution of p16 expression in the mammary epithelium. Due to the heterogeneity in morphology of the juxta-tumoral glands, we estimated p16 levels in normal-looking structures and lesion areas separately. Consistent with our gene expression analysis, we found that the percentage of p16 + cells was higher in normal-looking epithelium of Itgα6-deficient females, whereas this difference disappeared in the areas with lesions (Fig. 5C). Additionally, Western blot analysis with protein extracts from sorted LP of preneoplastic glands showed higher p16 levels in α6-deficient cells (Fig. 5D).

Furthermore, in agreement with previous studies, we found that, in Brca1/p53-deficient epithelium, a non-negligible fraction of p16 + cells was abnormally cycling, as shown by their Ki67 status (Fig. 5E; [40, 41]. Of interest, we found that the amount of cycling p16 + cells was reduced in Itgα6-deficient glands, predominantly in normal-looking epithelial structures, suggesting cell cycle misregulation in cells expressing p16 in preneoplastic glands (Fig. 5E).

The mode of action of the cell cycle inhibitor p16 involves the inhibition of the CDK4/6 kinase, resulting in hypo-phosphorylation of the retinoblastoma (Rb) protein, locking cells in a cell cycle arrest [42]. Consistent with a higher amount of non-cycling p16 + cells, we also detected a reduction in the proportion of cells expressing phosho-Rb in α6KO/Brca1p53-KO mutant mammary epithelium, particularly in the normal-looking ducts (Fig. 5F). Accordingly, the levels of phospho-Rb protein were reduced in luminal cells depleted of Itgα6 (Fig. 5D). Taken together, these results indicate that lack of Itgα6 favors the cell cycle inhibitor function of p16, leading to reduced proliferation in Brca1/p53 mutant preneoplastic glands.

## Discussion

Dysregulated integrin expression and function can impact tumor development at several levels: from the initial transformation of epithelial cells at tumor onset, to cancer cell migration and invasion through tissue boundaries and colonization of distant tissues. In breast cancer, Itgα6 has been associated with tumor cell invasion and metastasis [24]. However, its role in the early steps of tumorigenesis has not been investigated. We describe here the important contribution of Itgα6 to the formation of basal-like mammary tumors. Using a mouse model of aggressive breast cancer due to the induced deficiency of Brca1 and p53 in the mammary luminal epithelium, we show that deletion of *Itga6* in the tumor cells of origin, the luminal progenitors, impairs tumor formation through a mechanism involving: (i) inhibition of cell cycle progression associated to over-expression of p16 in pre-tumoral glands and (ii) interference with an EMT-like program normally occurring during the first stages of tumorigenesis triggered by Brca1/p53 loss.

We have chosen to examine the phenotype of induced loss of Itga6 in *Blg-Cre;Brca1*^*F/F*^*;Trp53*^*F/F*^ mice, a well-established model of basal-like tumors, mimicking features characteristic of human BRCA1-deficient breast cancer [14]. In these mice, the Blg promoter targets gene deletion in luminal progenitors, which are considered the cells of origin of Brca1-mutant basal-like tumors. Consistently with our previous work in homeostatic glands, we detected Blg-driven *Cre* expression and targeted gene deletion predominantly in ICAM1 + LP [23]. However, some contribution of the ICAM1- mature luminal cells to tumorigenesis i.e. providing mitogenic signals, cannot be excluded. The role of mature luminal cell transformation in the genesis of Brca1-deficient tumors would require further investigation and the use of promoters specifically targeting this cell population, such as ER or Prominin-1 promoters.

Although in many types of cancers, including breast, Itgα6 has been reported to promote tumorigenesis, its expression is anti-correlated with tumor progression and invasion in some types of leukemia and prostate cancer [43, 44]. Furthermore, Itgα6 has been categorized as a tumor suppressor in the gut, where its ablation, by disrupting hemidesmosomes, leads to intestinal epithelium detachment and inflammatory lesions that progress to colorectal carcinoma [27]. Therefore, the role of this integrin in tumorigenesis is pleiotropic and appears to be context dependent.

The integrin α6 subunit can associate with β1 and β4 to form α6β1 and α6β4 laminin-receptor dimers. While the β1-subunit is highly promiscuous, α6 is the only known partner of the β4 subunit. Accordingly, we found that Itgβ4 levels are reduced in luminal cells as well as in tumors in α6KO/Brca1p53-KO mice. In agreement with our results, deletion of the Itgβ4 signaling domain in a mouse model of Erbb2-induced mammary carcinoma resulted in impaired tumor formation [45]. Moreover, Itgβ1 is dispensable for tumor induction in the same mouse model, although its deletion leads to a delayed tumor onset and reduced tumor volume [46]. We have not detected significant changes in the cell surface levels of Itgβ1 in our α6KO/Brca1p53-KO mice. Therefore, our results suggest that the decreased levels of α6β4 (rather than those of α6β1) in the α6KO/Brca1p53-KO epithelium predominantly contribute to the observed phenotypes.

High expression of Itgα6 serves as a marker for isolating stem cells in different tissues [47]. We have previously shown that the simultaneous depletion of α3 and α6 integrins results in a significant perturbation of mammary gland development and function [23]. Depletion of Itgα6 alone led to mild mammary phenotype during lactation, while depletion of Itgα3 did not have significant effects. In particular, during pregnancy-associated alveologenesis, Itgα6 seems dispensable for the amplification of luminal progenitors [23]. On the contrary, here we found that, in the absence of Itgα6, expansion of the functional luminal progenitor population that precedes tumorigenesis in Brca1/p53-deficient mice is impaired, indicating that Itgα6 has different roles in normal physiology and tumor formation.

Itgα6 has also been shown to enrich for tumor initiating activity in breast and other tumor types [24]. In ER- breast tumors, cells with high Itgα6 expression display heightened tumorigenicity and self-renewal in vivo [48]. In the same vein, a subpopulation of CD24^+^Itgα6^+^Itgβ1^+^ cells with increased proliferation and enhanced tumor-forming ability has been found in mouse cell lines derived from p53^±^;Brca1-deficient tumors [49]. Itgα6 exists as two different cytoplasmic variants, α6A and α6B, generated by alternative splicing [50]. The Mercurio’s lab reported that the α6B isoform promotes tumorigenesis in human breast cancer cell lines, unlike the α6A isoform, which is dispensable for tumor formation [51, 52]. The *loxP* cassette used for *Itga6* deletion in our study includes the transmembrane and the cytoplasmic exons specific of each of the two splicing variants, leading to the lack of expression of both isoforms in the α6KO/Brca1p53-KO mice [27]. Identification of the isoform involved in tumor induction in our model warrants further investigation.

Previous studies using similar Brca1-deficient mouse models described aberrant mammary alveolar development and single cell RNAseq analysis revealed the existence, at the premalignant stage, of a molecular cell cluster resembling luminal alveolar cells typical of gestation in the homeostatic gland [35, 36]. Alveologenesis mimicry and lineage infidelity may significantly contribute to establishment of breast cancer [53]. We indeed found aberrant alveolar-like structures in the mutant preneoplastic glands associated to increased epithelial proliferation. However, these phenotypes were attenuated in the absence of Itgα6 and this was accompanied by the aforementioned reduction in luminal progenitor expansion. Notably, the clonogenic potential of luminal progenitors was reduced by *Itga6* deletion, potentially contributing to the delayed tumor onset observed in α6KO/Brca1p53-KO mice.

Partial epithelial-to-mesenchymal transition (pEMT) has been associated to Brca1-induced tumorigenesis [16, 17]. This process implies the existence of intermediate cellular states displaying both epithelial and mesenchymal (E/M) traits, that have been correlated to increased tumorigenicity in different types of cancer (reviewed in [54, 55]). Furthermore, a high frequency of cells co-expressing basal and luminal specific markers (K8 + /K14 + cells) has been associated with malignancy in human Brca1-mutation carriers and in tumor mouse models [39, 56]. In line with these data, we have detected an increased expression of basal and EMT-related genes in the luminal progenitors from juxta-tumoral tissue in Brca1p53-KO mice. This phenotype appears mitigated in the cells lacking Itgα6 expression suggesting that Itgα6 is required for the luminal-to basal switch and the concomitant triggering of a pEMT program in the Brca1/p53-deficient epithelium. Consistently, using a model of TNBC cells, Bierie et al. have shown the existence of Itgβ4^+^ cells residing in an intermediate E/M phenotypic state and displaying high tumorigenicity [57].

**Fig. 6:**
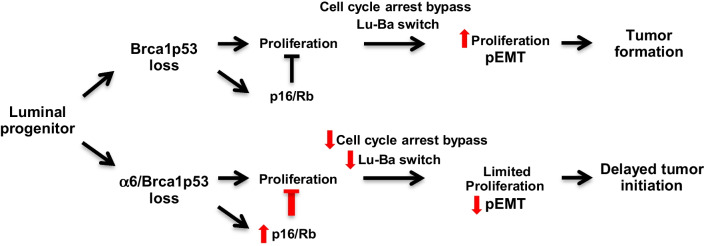
Model of the impact of Itgα6 loss in the formation of Brca1p53-deficient tumors. Loss of Brca1 and p53 in luminal progenitor cells induces epithelial proliferation that is, in first instance, counterbalanced by the concomitant activation of the p16/Rb pathway. With time and the plausible accumulation of DNA damage, a cell cycle arrest bypass occurs that, accompanied by the luminal-to-basal switch and pEMT, leads to tumor formation. In the Itgα6-deficient animals, overactivation of p16/Rb pathway in preneoplastic tissue restrains epithelial proliferation and limits luminal-to basal switch and pEMT, resulting in delayed tumor initiation

Of interest, in α6KO/Brca1p53-KO mice we have detected the overexpression of the cell cycle inhibitor p16, associated with hypo-phosphorylated Rb and a reduced proportion of cycling cells. Compared to other breast cancer types, basal-like invasive tumors display increased activation of the p16/Rb pathway [40]. Furthermore, single cell RNAseq analysis has recently revealed the presence of a p16-expressing luminal population in the pre-malignant tissue of the same Brca1/p53-deficient mice used in our study [41]. Suppression of Itgα6 has been shown to induce the expression of the cell cycle inhibitor p27 resulting in compromised cell cycle progression in a triple-negative breast cancer cell line [58]. In addition, a recent study reported that in the absence of Rb, Brca1p53-KO mice develop luminal ER + (instead of basal-like) tumors, indicating that Rb signaling is involved in the luminal-to-basal switch in Brca1/p53-deficient epithelium [59]. Therefore, the perturbation of the p16/Rb pathway at early stages of malignancy could provide a mechanistic explanation for the observed phenotypes (reduced proliferation, inhibition of luminal-to-basal switch) resulting in delayed tumorigenesis in α6KO/Brca1p53-KO mice (Fig. 6).

High Itgα6 levels are associated with poor survival in breast cancer and *ITGA6* expression is an independent prognosis factor in ER-negative breast cancer [60–63]. Furthermore, this integrin subunit contributes to breast cancer cell dissemination and metastasis [64, 65]. The Brca1p53-KO model used in this study is poorly metastatic, precluding the analysis of the involvement of Itgα6 in metastatic stages. However, we found that in advanced stages of tumorigenesis, Itgα6 appears dispensable for tumor growth, but its deletion affects tumor cell differentiation, presenting lower expression of basal markers, probably reflecting the inefficient induction of luminal/basal double-positive at the early steps of tumor formation. Importantly, our results have uncovered a critical role for Itgα6 in the first stages of breast cancer initiation, suggesting that its expression in luminal cells could be causally linked to tumor progression and malignant phenotype and as such represent a promising tool to predict the evolution of early breast lesions.

### Supplementary Information

Supplementary file

## Data Availability

Data sharing is not applicable to this article as no datasets were generated during the current study.

## Abbreviations

BM: Basement membrane
CC3: Cleaved-caspase-3
CDK: Cyclin-dependent kinase
DAPI: 4′6-Diamidino-2-phenylindole
ECM: Extracellular matrix
EdU: 5-Ethynyl-2′-deoxyuridine
ER: Estrogen receptor
FACS: Fluorescence-activated cell sorting
ICAM: Intercellular adhesion molecule-1
ITG: Integrin
K14, K5, K8: Cytokeratin 14, 5, 8
LM: Luminal mature
LP: Luminal progenitor
MEC: Mammary epithelial cell
pEMT: Partial epithelial-mesenchymal transition
PR: Progesterone receptor
RANKL: Receptor activator of nuclear factor kappa-B ligand
Rb: Retinoblastoma
RT-qPCR: Reverse-transcription-quantitative polymerase chain reaction
SEM: Standard error of the mean
α-SMA: α-Smooth-muscle actin
