# Supplementary material for: Alpha-6 integrin deletion delays the formation of Brca1/p53-deficient basal-like breast tumors by restricting luminal progenitor cell expansion
Source: Breast Cancer Res. 2024 Jun 4;26:91. doi: 10.1186/s13058-024-01851-4 (PMC11151721; doi:10.1186/s13058-024-01851-4)
Supplement: Supplementary file 1 — Supplementary file [file 13058_2024_1851_MOESM1_ESM.pdf]

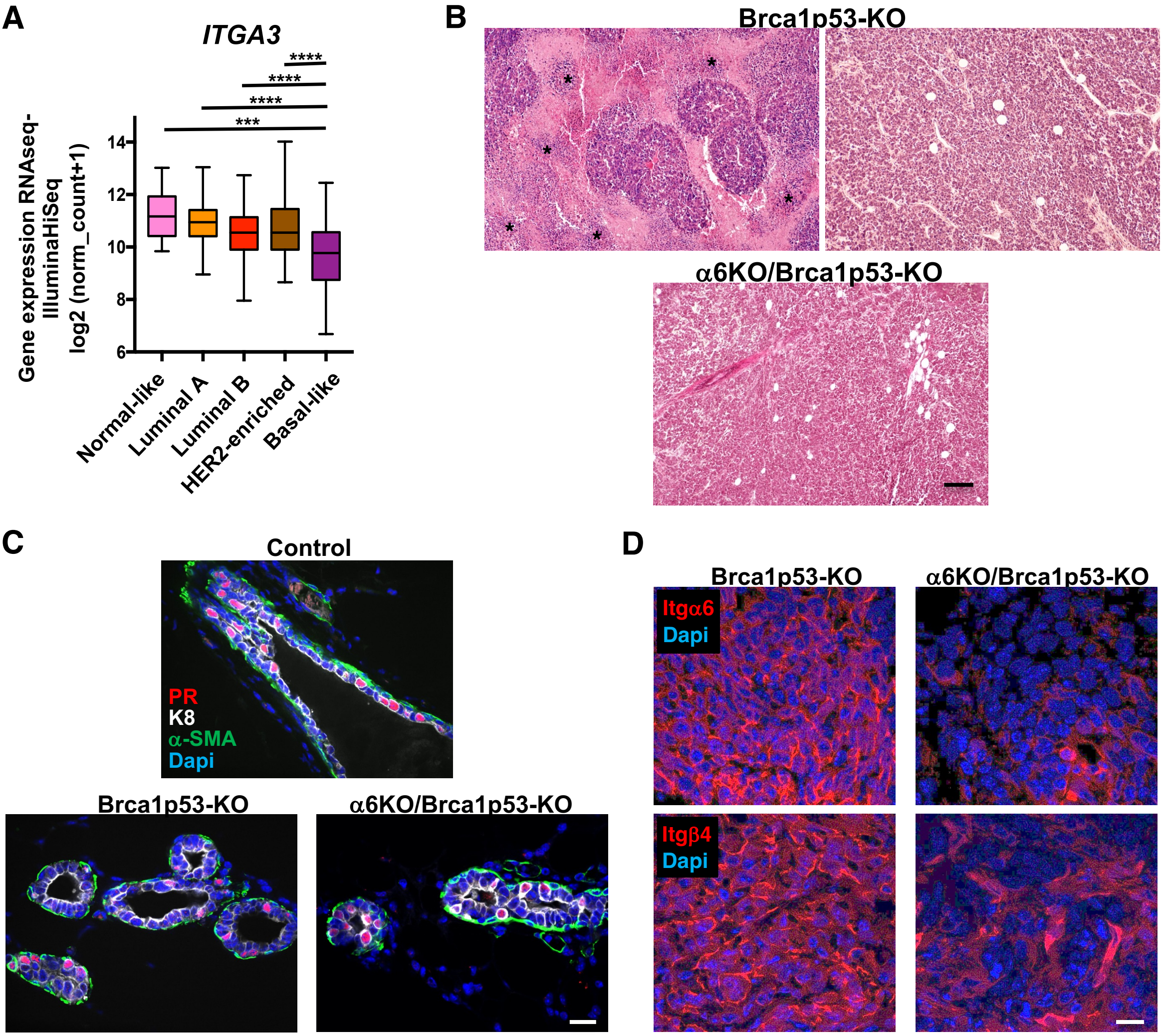

Additional File 1: Figure S1 Expression of laminin-binding integrins in breast tumors**. A** *In silico* analysis of *ITGA3* mRNA expression in human breast cancer subtypes classified by PAM50 signature: Normal-like (n = 8), Luminal A (n = 231), Luminal B (n = 127), HER2-enriched (n = 58), and basal-like (n = 98). ****P<0.0001. **B** Representative images of large tumors (Hematoxylin/eosin staining) showing the presence of large necrotic areas in large (> 1200 mm^3 volume) Brca1p53-KO tumors (stars in left image), that are not detected in the α6KO/Brca1p53-KO tumors of similar size. Bar, 100 μm. **C** Immunofluorescent staining of mammary glands from control (BlgCre-neg) and Brca1 mutant mice with anti-PR (red), anti-K8 (white) and anti α-SMA (green) antibodies. **D** Immunofluorescent staining with anti-α6-integrin (upper panels) and anti-β4-integrin antibodies (lower panels), both in red, showing the absence of these integrin subunits in most tumor cells. C, D: Nuclear DAPI staining is shown in blue. Bar, 20 μm.

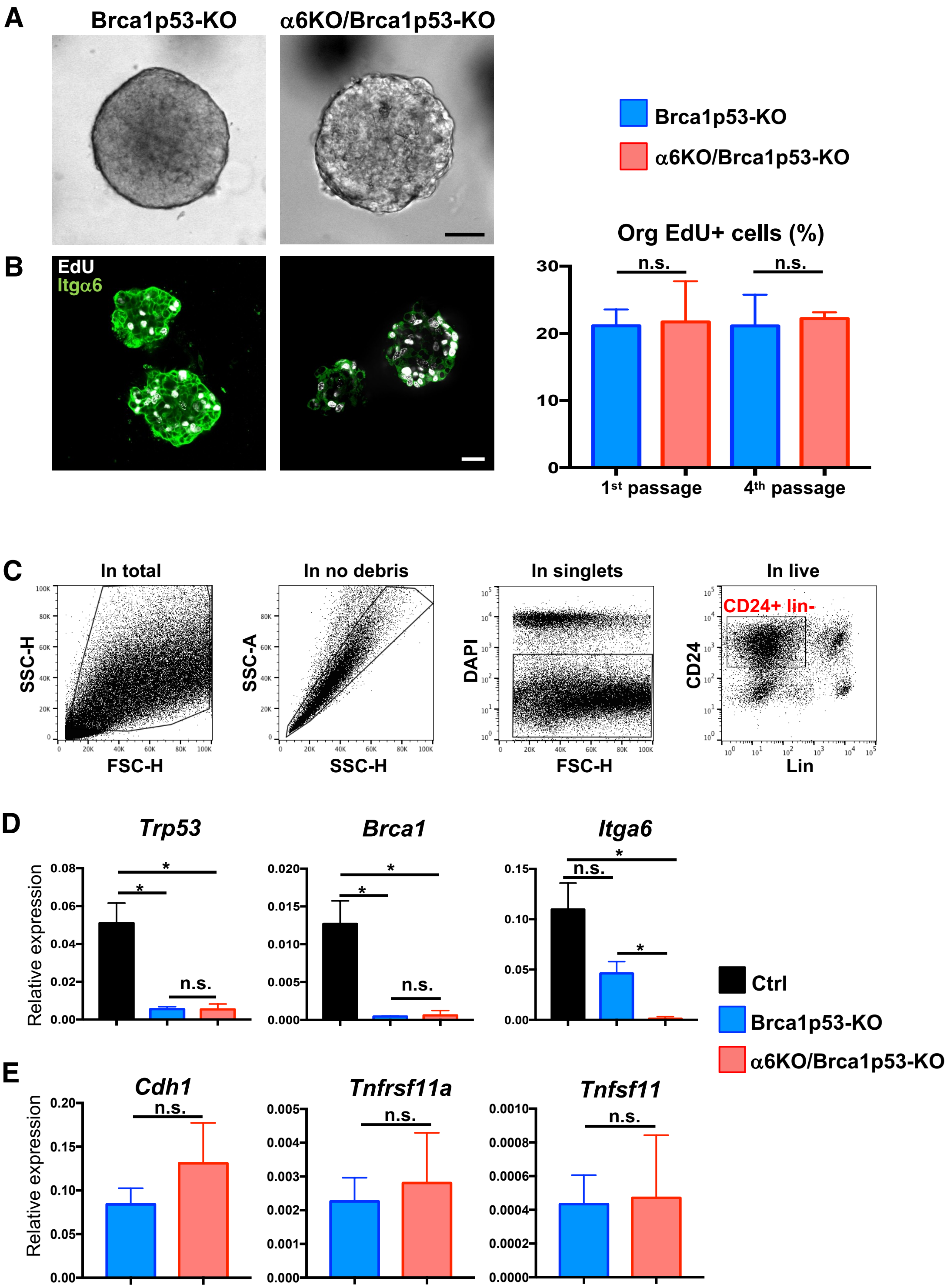

A
Brca1p53-KO
α6KO/Brca1p53-KO
Brca1p53-KO
α6KO/Brca1p53-KO
B
EdU
Itgα6
Org EdU+ cells (%)
n.s.
n.s.
30
20
10
0
1st passage
4th passage
C
In total
In no debris
In singlets
In live
SSC-H
FSC-H
SSC-A
SSC-H
DAPI
FSC-H
CD24
Lin
CD24+ lin-
D
Trp53
Brca1
Itga6
Relative expression
n.s.
*
Ctrl
Brca1p53-KO
α6KO/Brca1p53-KO
E
Cdh1
Tnfrsf11a
Tnfsf11
Relative expression
n.s.

Additional File 2: Figure S2 Effect of *Itga6* deletion in growth and differentiation of Brca1/p53-deficient tumors. **A** Bright field images of tumor organoids after 10 days in culture. **B** Immunofluorescent staining of tumor organoids cultured for 10 days and incubated with EdU during 2 hours before fixation: EdU detection (white), anti-Itgα6 antibody (green). Scale bar: 20 μm (A, B). The graph shows the percentage of EdU+ cells after 1 and 4 passages (mean±SEM) in n = 3 tumors per group. n.s., non-significant. **C** FACS gate strategy for isolation of CD24+ lin- cells from tumors. **D, E** RT-qPCR analysis of the CD24+ Lin-negative tumor cell population in five Brca1p53-KO and five α6KO/Brca1p53-KO animals (mean±SEM). In D, gene expression levels of *Trp53*, *Brca1* and *Itga6* were compared to those obtained in sorted luminal cells from a control (BglCre-neg;Brca1$^{F/F}$;Trp53$^{F/F}$) female (n = 3). *P < 0.05. n.s., non-significant.

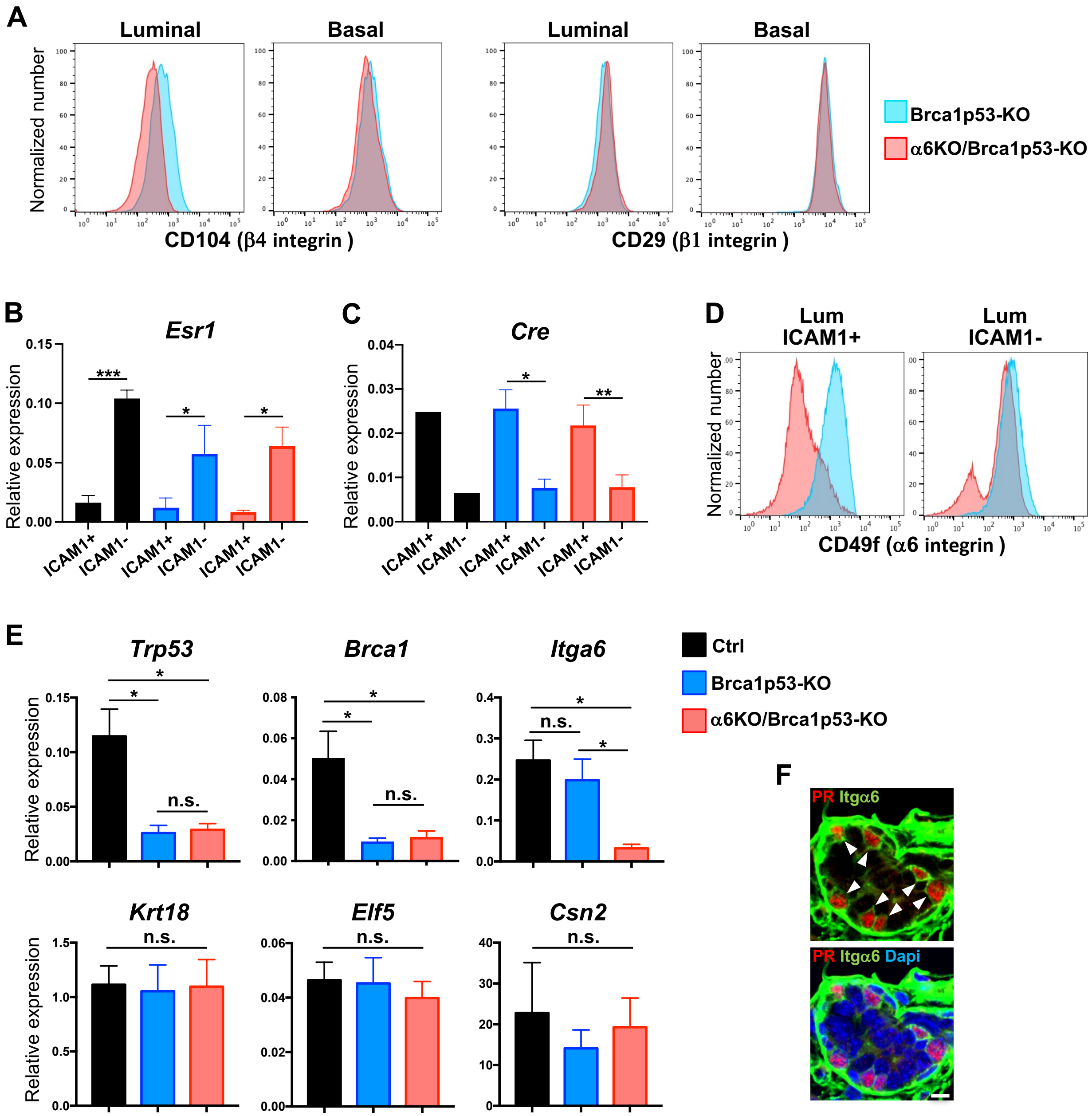

A
Luminal
Basal
Luminal
Basal
Normalized number
CD104 (β4 integrin)
CD29 (β1 integrin)
Brca1p53-KO
α6KO/Brca1p53-KO
B
Esr1
C
Cre
Relative expression
ICAM1+
ICAM1-
D
Lum ICAM1+
Lum ICAM1-
CD49f (α6 integrin)
E
Trp53
Brca1
Itga6
Krt18
Elf5
Csn2
n.s.
Ctrl
Brca1p53-KO
α6KO/Brca1p53-KO
F
PR Itgα6
PR Itgα6 Dapi

Additional File 3: Figure S3 Analysis of the preneoplastic glands of Brca1/p53-deficient mice. **A** Histograms showing the expression of β4 and β1-integrins in luminal and basal mammary cells isolated from 5-month-old Brca1p53-KO and α6KO/Brca1p53-KO mice as described in Fig. 3C. **B,** RT-qPCR analysis of *Esr1* expression in ICAM1+ and ICAM1- luminal cells from control (n = 4), Brca1p53-KO (n = 5) and α6KO/Brca1p53-KO (n = 5) glands. **C,** RT-qPCR analysis of *Cre* expression in ICAM1+ and ICAM1- luminal cells from Brca1p53-KO and α6KO/Brca1p53-KO glands (5 animals per group). Expression in a Blgcre+ control mice is shown for comparison. **D** Histograms showing expression of α6-integrin in ICAM1+ and ICAM1- luminal cells from Brca1p53-KO and α6KO/Brca1p53-KO glands separated as described in Fig. 3D. **E** RT-qPCR analysis of the ICAM1+ luminal progenitor cells from control, Brca1p53-KO and α6KO/Brca1p53-KO glands (5 animals per group). **F** Immunofluorescent staining of a α6KO/Brca1p53-KO gland with anti-α6-integrin (green) and anti-PR antibodies (red), showing expression of the integrin in PR+ cells. Nuclear DAPI staining is shown in blue. Arrowheads show cells co-expressing α6-integrin and PR. Scale bar: 12 μm. In **B**, **C** and **E**, $^{*}P < 0.05$, $^{**}P < 0.01$, $^{***}P < 0.001$. n.s., non-significant.

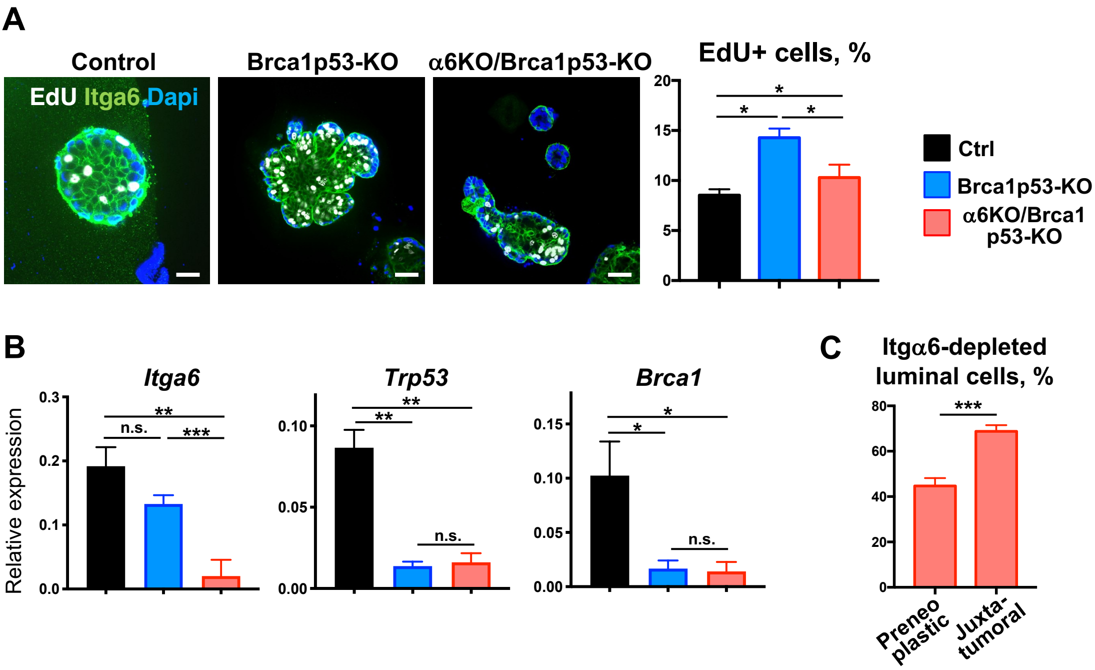

Additional File 4: Figure S4 Analysis of juxta-tumoral glands of Brca1-deficient mice. **A** Immunofluorescent staining of organoids obtained from mammary epithelial fragment cultured for 10 days and incubated with EdU during 2 hours before fixation: EdU detection (white), anti-Itgα6 antibody (green). Bar, 20 μm (left panel), 40 μm (central and right panels). The graph shows the percentage of EdU+ cells (mean±SEM) in three animals per group. **B** RT-qPCR analysis of the ICAM1+ luminal progenitor cells from control (n = 3), Brca1p53-KO (n = 5), and α6KO/Brca1p53-KO (n = 5) juxta-tumoral glands. **C** Percentage of Itgα6-negative luminal cells analyzed by Flow cytometry (as shown in Fig. 3C and 4C) in preneoplastic (n = 7) and juxta-tumoral (n = 4) glands of α6KO/Brca1p53-KO mice. In **A**-**C**, *P < 0.05, **P < 0.01, ***P < 0.001. n.s., non-significant.

Additional File 5: Table S1 - Antibodies used for Immunofluorescence (IF) and Western Blot (WB)

Primary Antibodies	Source	Identifier	Dilution-IF	Dilution-WB
Mouse anti-β-actin	Sigma	A2228		1:5000
Rabbit anti-cleaved caspase3	Cell Signaling Technology	9664	1:100	
Rabbit anti-keratin 5	Covance	PRB-160P	1:300	
Rat anti-K8	Developmental Studies Hybridoma Bank	TROMA-I	1:300	
Rabbit anti-keratin 14	Abcam	Ab181595	1:200	1:2000
Rabbit anti-Ki67	Invitrogen	MA5-14520	1:150	
Rabbit anti-Ki67	Eurobio Scientific	API3156AA	1:100	
Rat anti-Itgα6	BD-Pharmingen	555734	1:100	
Rat anti-Itgβ4	BD-Pharmingen	553745	1:100	
Rabbit anti-p16	Abcam	Ab211542	1:100	1:1000
Rabbit anti-PR	SantaCruz	SC7208	1:200	
Rabbit anti-Phospho-Rb (S807/811)	Cell Signaling Technology	8516	1:100	1:1000
Mouse anti-α-SMA	Sigma-Aldrich	A2547	1:200	

Secondary Antibodies	Source	Identifier	Dilution - IF	Dilution - WB
Goat anti-mouse AlexaFluor-coupled to different fluorochromes (Cy3, Cy5, A488)	Invitrogen-Thermo Fisher Scientific	A10521, A10524, A11001	1:500	
Goat anti-rat AlexaFluor-coupled to different fluorochromes (Cy3, Cy5, A488)	Invitrogen-Thermo Fisher Scientific	A10522, A10525, A11006	1:500	
Goat anti-rabbit AlexaFluor-coupled to different fluorochromes (Cy3, Cy5, A488)	Invitrogen-Thermo Fisher Scientific	A10520, A10523, A11034	1:500	
Anti-mouse HRP-linked	Cell Signaling Technology	7076		1:3000
Anti-rabbit HRP-linked	Cell Signaling Technology	7074		1:3000

Additional File 6: Table S2- Sequences of the primers used for RT-qPCR

***Gene* -primer**	**Primer sequence (5'-3')**
Brca1 -s	CTGAGGGCATAAGAAACATTG
Brca1 -as	GGCTCCACACACACATTTGA
Cdh1 -s	ATCCTCGCCCTGCTGATTC
Cdh1 -as	ACCACCGTTCTCCTCCGTA
Cdkn2a -s	CCCCGATTCAGGTGATGATG
Cdkn2a -as	ACCGTAGTTGAGCAGAAGAG
Csn2 -s	CCTCTGAGACTGATAGTATTT
Csn2 -as	TGGATGCTGGAGTGAACTTTA
Elf5 -s	CCAACGCATCCTTCTGTGAC
Elf5 -as	AGGCAGGGTAGTAGTCTTCA
Esr1 -s	CTGGACAGGAATCAAGGTAAA
Esr1 -as	GAGGCACACAAACTCTTCTC
Fn1 -s	GAAGAGCCCTTACAGTTCCA
Fn1 -as	GTGCCTCCACTATGATGTTG
Gapdh -s	CCAATGTGTCCGTCGTGGATC
Gapdh -as	GTTGAAGTCGCAGGAGACAAC
Krt18 -s	CCTTGCCGCCGATGACTTTA
Krt18 -as	CAGCCTTGTGATGTTGGTGT
Krt5 -s	GACCAGTCAACATCTCTGTC
Krt5 -as	TGCCAACACCAATGCTGCTG
Snai2 -s	GATGCCCAGTCTAGGAAATC
Snai2 -as	CCCAGTGTGAGTTCTAATGT
Trp53 -s	AGGGTCGAGACACAATCCTC
Trp53 -as	GCAGAGACCTGACAACTATCA
Vim -s	CCAAGCAGGAGTCAAACGA
Vim -as	TAAGGGCATCCACTTCACAG

Other primers were purchased from SABiosciences/Qiagen

Figure S5

A

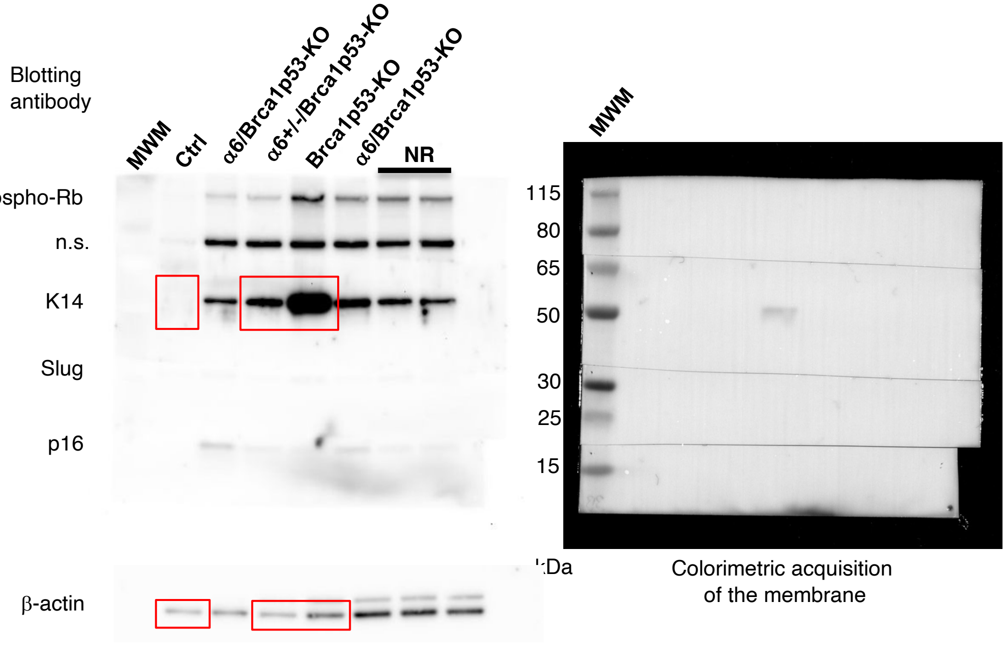

Blotting antibody
MWM
Ctrl
α6/Brca1p53-KO
α6+/-/Brca1p53-KO
Brca1p53-KO
α6/Brca1p53-KO
NR
Phospho-Rb
n.s.
K14
Slug
p16
β-actin
MWM
115
80
65
50
30
25
15
kDa
Colorimetric acquisition of the membrane

B

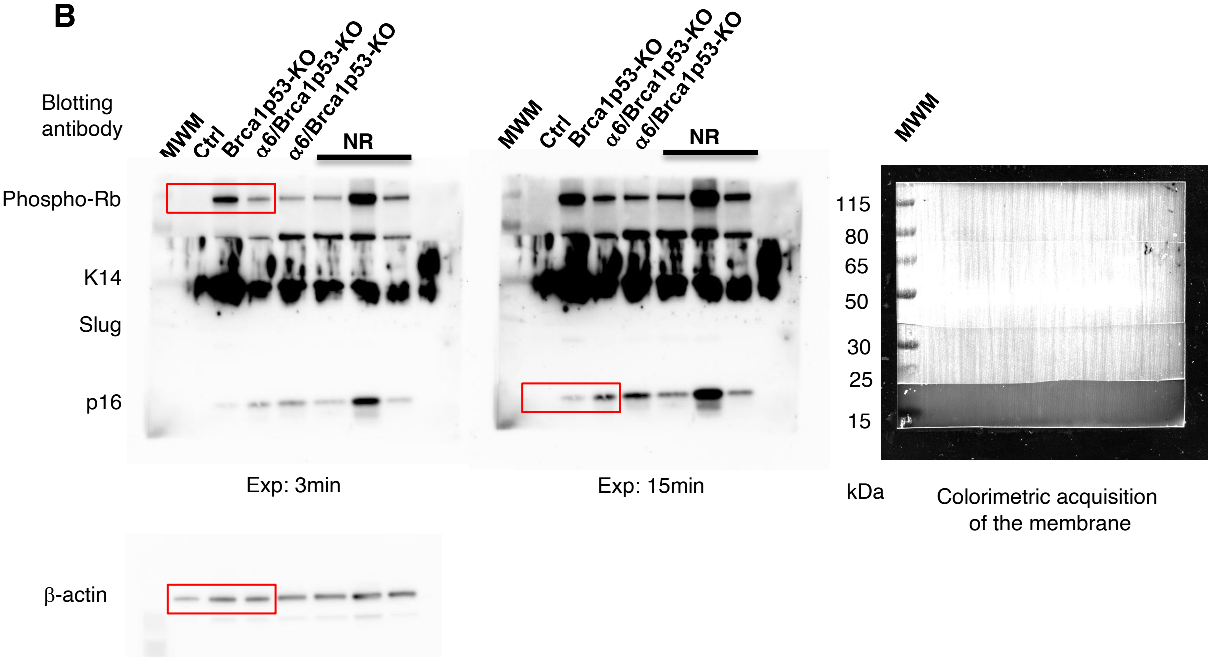

Blotting antibody
MWM
Ctrl
Brca1p53-KO
α6/Brca1p53-KO
α6/Brca1p53-KO
NR
Phospho-Rb
K14
Slug
p16
Exp: 3min
Exp: 15min
β-actin
MWM
115
80
65
50
30
25
15
kDa
Colorimetric acquisition of the membrane

Additional File 7: Figure S5 Whole images of the presented Western blots. The membranes were fragmented before incubation with the antibodies indicated at the left of the blot image. A colorimetric acquisition of the entire membrane is shown in the right panels. Red squares delineate the images presented in Fig. 4G and 5D (A, B). **A** The membrane was blotted, among other antibodies, with K14, presented in Fig. 4G. **B** The membrane was blotted, among other antibodies, with phospho-Rb and p16, presented in Fig. 5D (different exposures are shown). After stripping, the membrane fragment corresponding to 50-65 kDa, was re-blotted for β-actin detection (lower panels in A and B). MWM: molecular weight markers. NR: non relevant samples analyzed in the same blot; n.s., non-specific band.
